# Supplementary material for: Streaming Batch Eigenupdates for Hardware Neural Networks
Source: Front Neurosci. 2019 Aug 6;13:793. doi: 10.3389/fnins.2019.00793 (PMC6691093; doi:10.3389/fnins.2019.00793)
Supplement: Supplementary file 1 [file Data_Sheet_1.docx]

Supplementary Material

# Taylor Expanded SBE Algorithm

If we assume that the SBE algorithm has been running for some time such that $\mathbf{X}^{i,j}$ and $\boldsymbol{\Delta}^{i,j}$ are already unit vectors and the learning rate is small (as could be in the case of large *j*, leading to a small learning rate $\xi$), we could formulate the algorithm first with an explicit normalization and then expand this in a Taylor series:

$$\mathbf{X}^{i,j+1}=\frac{\mathbf{X}^{i,j}+\xi\mathbf{x}^{i,j}\left( \boldsymbol{\delta}^{i,j}\cdot\boldsymbol{\Delta}^{i,j} \right)}{(\mathbf{(X}^{i,j}+\xi\mathbf{x}^{i,j}(\boldsymbol{\delta}^{i,j}\cdot\boldsymbol{\Delta}^{i}))\cdot(\mathbf{X}^{i,j}+\xi\mathbf{x}^{i,j}(\boldsymbol{\delta}^{i,j}\cdot\boldsymbol{\Delta}^{i,j})))^{\frac{1}{2}}}$$

For small values of $\xi$, the expansion of the singular vector is:

$$\mathbf{X}^{i,j+1}=\mathbf{X}^{i,j}\left( 1-\xi\boldsymbol{\delta}^{i,j}\cdot\boldsymbol{\Delta}^{i,j} \mathbf{x}^{i,j}\cdot\mathbf{X}^{i,j} \right)+\xi\mathbf{x}^{i,j}(\boldsymbol{\delta}^{i,j}\cdot\boldsymbol{\Delta}^{i,j})$$

Which naturally implies the other values:

$$\boldsymbol{\Delta}^{i,j+1}=\boldsymbol{\Delta}^{i,j}\left( 1-\xi\boldsymbol{\delta}^{i,j}\cdot\boldsymbol{\Delta}^{i,j} \mathbf{x}^{i,j}\cdot\mathbf{X}^{i,j} \right)+\xi\boldsymbol{\delta}^{i,j}(\mathbf{x}^{i,j}\cdot\mathbf{X}^{i,j})$$

$${\sigma^{i,j+1}}={\sigma^{i,j}}(1-\xi)+\xi\boldsymbol{\delta}^{i,j}\cdot\boldsymbol{\Delta}^{i,j} \mathbf{x}^{i,j}\cdot\mathbf{X}^{i,j}$$

The necessary hardware values that need to be calculated and stored are the scalars $\mathbf{x}^{i,j}\cdot\mathbf{X}^{i,j}, \boldsymbol{\delta}^{i,j}\cdot\boldsymbol{\Delta}^{i,j}$, and ${\sigma^{i,j}}$ along with the necessary update vectors to the singular vectors. With suitable hardware, the MAC, multiplication, and subtraction operations can occur in parallel.
